# Does Nitrate Supplementation Improve Muscle Strength, Power, and Sprint Performance in Females? A Systematic Review and Meta-Analysis

**DOI:** 10.3390/life15091425

**Published:** 2025-09-11

**Authors:** Fanhao Meng, Yuhang Liu, Bopeng Qiu, Juan Li

**Affiliations:** 1 School of Strength and Conditioning Training, Beijing Sport University, Beijing 100084, China; fanhaom0712@163.com (F.M.); qiubopeng@bsu.edu.cn (B.Q.); 2 Exercise and Sport Nutrition Lab, Department of Kinesiology and Sport Management, Texas A&M University, College Station, TX 77843-4243, USA; yhliu@tamu.edu; 3 Division of Sports Science & Physical Education, Tsinghua University, Beijing 100084, China; 4 School of Physical Education and Sports, Central China Normal University, Wuhan 430079, China

**Keywords:** inorganic nitrate, beetroot juice, power, muscle strength, sprint performance, female athletes

## Abstract

Background: Inorganic nitrate (NO_3_^−^) may enhance muscle contractility and blood flow via nitric oxide production, offering potential ergogenic benefits. However, most studies have focused on males, and its effects in females during high-intensity, short-duration exercise remain unclear. Objective: This review assessed the acute effects of nitrate supplementation on muscle strength, power, and sprint performance in healthy females. Methods: A comprehensive literature search was conducted in PubMed, Web of Science, Scopus, SPORTDiscus, and Cochrane CENTRAL, from inception to July 2025. Randomized controlled trials (RCTs) that examined the effects of oral inorganic nitrate (e.g., beetroot juice or nitrate salts) in healthy females were included. Eligible studies involved (i) healthy women aged 18–30, (ii) nitrate supplementation vs. placebo, and (iii) outcome measures of muscle strength (e.g., MVC), power (e.g., countermovement jump height, peak power), or sprint performance (e.g., sprint time or repeated sprint ability). Studies were excluded if they did not report sex-specific results or lacked relevant physical performance outcomes. Random-effects meta-analyses were conducted for each outcome. Results: Nitrate supplementation had small, non-significant effects on muscle strength (SMD = 0.10, 95% CI: −0.10 to 0.30, *p* > 0.05) and sprint performance (SMD = 0.14, 95% CI: −0.13 to 0.41, *p* > 0.05). A statistically significant, small-to-moderate improvement was observed in power (SMD = 0.38, 95% CI: 0.06 to 0.69, *p* < 0.05). Sensitivity analyses confirmed robustness of the power result. The certainty of evidence ranged from low to moderate based on the GRADE assessment. Conclusions: Inorganic nitrate supplementation may modestly enhance power in healthy females but does not appear to significantly improve muscle strength or sprint performance. These findings highlight the importance of sex-specific research in sports nutrition to guide targeted supplementation strategies for female athletes.

## 1. Introduction

In recent years, nutritional supplements have gained increasing attention as strategies to optimize athletic performance. Beyond training adaptations, specific nutrients may enhance energy metabolism, muscle function, and fatigue resistance, thereby offering performance benefits that exceed those achieved by training alone [1,2]. Among the most extensively studied supplements, inorganic nitrate (NO_3_^−^) has emerged as a promising ergogenic aid due to its involvement in the nitric oxide (NO) pathway, which regulates blood flow and metabolism [3,4].

NO_3_^−^ is naturally abundant in green leafy and root vegetables, including beetroot, spinach, lettuce, and celery [5]. Beetroot juice (BRJ), with its high nitrate content, has become the most common form of supplementation and is widely used in pre-training and pre-competition nutritional strategies [5]. Following ingestion, dietary nitrate enters the entero-salivary-gastric circulation, where it is reduced to nitrite (NO_2_^−^) by anaerobic bacteria in the oral cavity, and subsequently converted to NO under hypoxic or acidic conditions [6]. This NO_3_^−^–NO_2_^−^–NO pathway functions independently of the nitric oxide synthase (NOS) and may be particularly relevant during high-intensity or oxygen-limited exercise [7]. As a key signaling molecule [8], NO plays a crucial role in vasodilation [9], skeletal muscle blood flow [10], mitochondrial efficiency, and excitation–contraction coupling, thereby supporting oxygen delivery and energy supply, muscle performance [11,12]. Nevertheless, findings regarding their effects on muscle strength and power remain inconsistent, suggesting the need for further targeted investigations [13].

Early research on nitrate supplementation, particularly BRJ, focused on aerobic endurance exercise at moderate to submaximal intensities, such as cycling [14,15,16,17] and distance running [18,19,20,21,22]. While individual studies produced mixed results, several systematic reviews and meta-analyses have consistently demonstrated that nitrate supplementation can reduce oxygen consumption and energy expenditure, improve exercise economy, and prolong time to exhaustion [23,24,25,26,27]. More recently, attention has shifted to high-intensity, short-duration, neuromuscularly demanding activities [28,29,30,31,32,33], which rely primarily on the phosphocreatine (PCr) energy system and recruit large numbers of type II fast-twitch muscle fibers [34]. For instance, its effects on power, maximal strength, and sprint performance have become focal points in current investigations [33].

Power—the ability to produce maximal mechanical output in minimal time—is critical for performing sport-specific actions including jumping, sprinting, and change in direction, and is a key determinant of athletic success [35,36]. While resistance training promotes strength and power through neural and structural adaptations, explosive movements are often limited by PCr depletion and calcium handling impairments [37]. Some studies have reported that nitrate supplementation can attenuate PCr depletion, enhance calcium reuptake, and improve excitation–contraction coupling. These effects are thought to involve both improved mitochondrial efficiency, which reduces the rate of PCr utilization, and enhanced sarcoplasmic reticulum Ca^2+^ handling that facilitates muscle contraction and relaxation [11,38]. While increased muscle blood flow may play a role, intramuscular mechanisms appear particularly important in supporting force output and endurance capacity during repeated high-intensity efforts [32,39,40]. Although several trials have demonstrated improvements in peak power output (Pmax), number of repetitions, and short-duration sprint performance following nitrate supplementation, findings remain inconsistent. Some studies have shown significant positive effects [41,42,43], while others reported no difference or highly variable individual responses [28,40,44]. Variability may be influenced by training status, supplementation dose and timing, or outcome measures.

A critical limitation of current literature is the underrepresentation of female participants. Most studies have been conducted in males or mixed cohorts, limiting sex-specific insights [45]. Yet females differ from males in several physiological domains, including hormone profiles, skeletal muscle composition, NO bioavailability, and fatigue recovery mechanisms [46,47]. The anabolic effects of testosterone result in greater muscle mass in males, which not only influences the response to supplementation, but may also affect the storage and utilization efficiency of nitrate and nitrite within the muscle tissue [48]. Conversely, some evidence suggests that females may experience greater increases in plasma NO_2_^−^ following nitrate ingestion, potentially translating into performance gains in explosive tasks such as jumping or sprinting [31,49,50]. However, other findings indicate a weaker or even null ergogenic effect in females under certain conditions [51,52,53,54,55].

With the rising participation of women in competitive and strength-based sports, reliance on male-centric data to guide nutritional strategies is increasingly inadequate. Addressing this gap is crucial for both scientific progress and applied practice. Therefore, this systematic review and meta-analysis specifically examines the effects of inorganic nitrate supplementation on muscle strength, power, and sprint performance in female athletes. This study aims to provide robust, sex-specific evidence for practitioners and help guide future research into sex-related responses to nitrate supplementation.

## 2. Methods

This meta-analysis adhered to the PRISMA 2020 guidelines (Preferred Reporting Items for Systematic Reviews and Meta-Analyses) to ensure transparency and completeness in reporting [56]. In line with open science practices and to promote reproducibility, the review protocol was prospectively registered on the PROSPERO database (https://www.crd.york.ac.uk/prospero/ (accessed on 17 July 2025) under registration ID: CRD420251106581).

### 2.1. Eligibility Criteria

The inclusion and exclusion criteria were developed according to the PICOS framework (Participanst, Intervention, Comparison, Outcomes, Study design) to ensure that all included studies addressed clearly defined populations, interventions, comparators, outcomes, and methodological rigor [57], as follows: Participanst (P): Eligible studies included healthy female participants, typically aged between 18 and 35 years, regardless of athletic status. Participants could be recreationally active, physically trained, or competitive athletes. Studies that included mixed-sex samples were considered only if sex-specific data for females were reported separately. Animal studies and non-human trials were excluded during the title and abstract screening phase. Intervention (I): Studies were required to evaluate the effects of oral inorganic nitrate supplementation—most commonly in the form of beetroot juice—administered acutely (e.g., single dose 2–3 h pre-exercise). There were no restrictions on dosage or delivery form, but the intervention protocol had to be clearly described, including the amount of nitrate provided and timing relative to performance testing. Comparison (C): Eligible studies had to include a placebo or non-supplemented control group under equivalent experimental or training conditions. In multiarm trials, comparisons between the nitrate group and a clearly defined placebo or control group were required. Outcomes (O): Studies were eligible if they re-ported at least one quantitative outcome related to physical performance, specifically focused on domains such as muscle strength (e.g., maximal voluntary isometric con-traction or isokinetic peak torque), power (e.g., countermovement jump height, rate of torque development, or peak power), or sprint performance (e.g., linear sprint time over 10–30 m or repeated sprint ability). Studies were included regardless of whether outcomes were measured in laboratory or sport-specific field tests, as long as the methodology and performance metrics were clearly described and extractable. Study design (S): Only randomized controlled trials (RCTs) or randomized crossover trials, published in peer-reviewed English language journals, were eligible for inclusion.

Studies were excluded if they met any of the following conditions: (i) No female-specific data were reported in mixed-sex trials; (ii) Use of multi-ingredient supplements without isolating the effect of nitrate; (iii) Absence of a placebo or control group; (iv) Lack of relevant performance outcomes or outcomes not extractable; (v) Non-original studies such as reviews, conference abstracts, theses, or protocols; (vi) Full text or critical data unavailable even after author contact.

### 2.2. Data Sources and Search Strategy

A comprehensive search of the literature was performed using five electronic databases: PubMed, Scopus, Web of Science, Cochrane CENTRAL, and SPORTDiscus, aiming to identify randomized trials that examined the effects of inorganic nitrate supplementation on muscle strength, explosive power, or sprint performance in female participants. To enhance the completeness of the search, additional strategies were employed, including manual screening of reference lists from all included articles, forward citation tracking through Google Scholar, and the use of the “Similar Articles” function in MEDLINE and Embase. Only peer-reviewed full-text publications in English were considered eligible. The search was independently conducted by two reviewers (F.M. and J.L.), covering studies published from database inception to 17 July 2025, with no restrictions applied to the year of publication. Boolean phrases and keywords used are detailed in Supplementary Table S2.

### 2.3. Study Selection and Screening Process

All retrieved records were first manually de-duplicated by an independent reviewer (F.M.) using EndNote X9 (Clarivate Analytics, Philadelphia, PA, USA). The resulting unique records were then screened independently by two reviewers (F.M. and J.L.) following the predefined inclusion and exclusion criteria. Title and abstract screening was performed to identify studies for full-text review. Any disagreements during this phase were resolved through discussion, and if needed, a third reviewer (Y.L.) was consulted to reach a consensus. Full-text articles were subsequently assessed in the same manner by the two reviewers to confirm final eligibility, with the same conflict resolution process applied when necessary. In addition, two supplementary approaches were used to identify potentially missed studies: (i) reviewing the reference lists of existing systematic reviews on related topics, and (ii) leveraging the reviewers’ expertise to locate relevant studies not captured in the initial database search.

### 2.4. Data Extraction and Transformation

Two reviewers (F.M. and J.L.) independently extracted data from each eligible study, and a third reviewer (B.Q) verified the accuracy and completeness of the extracted information. All data were compiled and managed in Microsoft Excel^®^ (Microsoft Corporation, Redmond, WA, USA) using a standardized extraction template. The following variables were collected: (i) Study characteristics: first author, year of publication, country, sample size, participant sex (female-specific), age, training status, and sport discipline; (ii) Intervention details: form and dosage of inorganic nitrate (e.g., beetroot juice), duration and timing of supplementation and control condition; (iii) Performance outcomes: measures of muscle strength (e.g., maximal voluntary isometric contraction, isokinetic peak torque), power (e.g., countermovement jump height, peak power, rate of torque development), and sprint performance (e.g., 10–30 m sprint time, repeated sprint performance); (iv) Study design and other relevant information: study type (randomized controlled or crossover trial), performance test timing (pre- and post-intervention), and any reported adverse effects. When outcome data were only available in graphical format, numerical values were estimated using WebPlotDigitizer (version 4.1; https://automeris.io/WebPlotDigitizer/ (accessed on 21 July 2025)) [58].

### 2.5. Risk of Bias and Certainty Assessment

The risk of bias for each included study was assessed using the Cochrane Risk of Bias 2.0 (RoB 2.0) tool [59], which evaluates five core domains: (i) The randomization process, (ii) Adherence to intended interventions, (iii) Completeness of outcome data, (iv) Measurement of outcomes, and (v) Selection of reported results. Two reviewers (F.M. and J.L.) independently performed the assessments, and any disagreements were resolved through discussion with a third reviewer (B.Q.) to reach consensus. A traffic light plot and weighted summary figure of the risk of bias across included studies were generated using the robvis visualization tool (https://mcguinlu.shinyapps.io/robvis/ (accessed on 24 July 2025)) [60].

The Grading of Recommendations Assessment, Development and Evaluation (GRADE) approach was used to assess the certainty of evidence across outcomes, with ratings categorized as high, moderate, low, or very low [61]. The GRADE assessment was conducted by one reviewer (F.M.) and independently verified by a second reviewer (Y.L.).

### 2.6. Statistical Analysis

All meta-analyses were performed using a random-effects model to accommodate heterogeneity across studies. Effect sizes were expressed as standardized mean differences (SMD), specifically calculated using the Standardized Mean Change with Correlation (SMCC) method, which is suitable for crossover or paired pre–post designs. This approach accounts for within-subject correlations, and is considered more precise than traditional change score methods for repeated-measures data [62], with a default correlation coefficient of r = 0.7, consistent with commonly recommended values for repeated measures outcomes [63,64]. A sensitivity analysis was conducted to assess the impact of varying r assumptions (range: 0.5–0.9) on the robustness of the pooled results.

When data were available only in graphical format, numerical values were extracted using WebPlotDigitizer (version 4.1). For studies that reported standard errors instead of standard deviations, *SD* was estimated using the formula:

(1) $$SD\;=\;SE\;\times \;\sqrt{n}\;$$

Due to variations in measurement units or test protocols across studies, SMD was consistently adopted as the summary effect size. To improve the consistency of outcome categorization across studies, countermovement jump (CMJ) and peak power were combined and analyzed together as a single construct representing lower-limb power. This decision was based on their similar physiological basis (i.e., reliance on rate of force development and neuromuscular output), and their common usage in the literature as interchangeable or complementary markers of power [35]. Effect sizes were interpreted using conventional thresholds, with 0.2 considered small, 0.5 moderate, and 0.8 large, as recommended in previous guidelines [65].

Heterogeneity was assessed using the *I*^2^ statistic, with the following interpretation: 0–40% (might not be important), 30–60% (moderate), 50–90% (substantial), and 75–100% (considerable heterogeneity). Additionally, the Jackson method was used to compute tau^2^ (τ^2^), tau (τ), and their 95% confidence intervals (CIs) [66].

Due to the limited number of studies per outcome (*n* ≤ 10), publication bias was not formally assessed using funnel plots or Egger’s regression test [57].

All analyses and visualizations were conducted using R (version 4.2.0) with the meta and metafor packages. Statistical significance was set at *p* < 0.05, while values between 0.05 and 0.10 were interpreted as suggestive of a trend.

## 3. Results

### 3.1. Literature Search

A total of 1041 records were initially retrieved through database searches, including PubMed (*n* = 199), Web of Science (*n* = 139), Scopus (*n* = 117), SPORTDiscus (*n* = 475), and Cochrane CENTRAL (*n* = 111). After removing 311 duplicate entries, 730 unique records remained for title and abstract screening. Based on predefined eligibility criteria, 705 records were excluded at this stage due to irrelevance to the topic.

**Figure 1:**
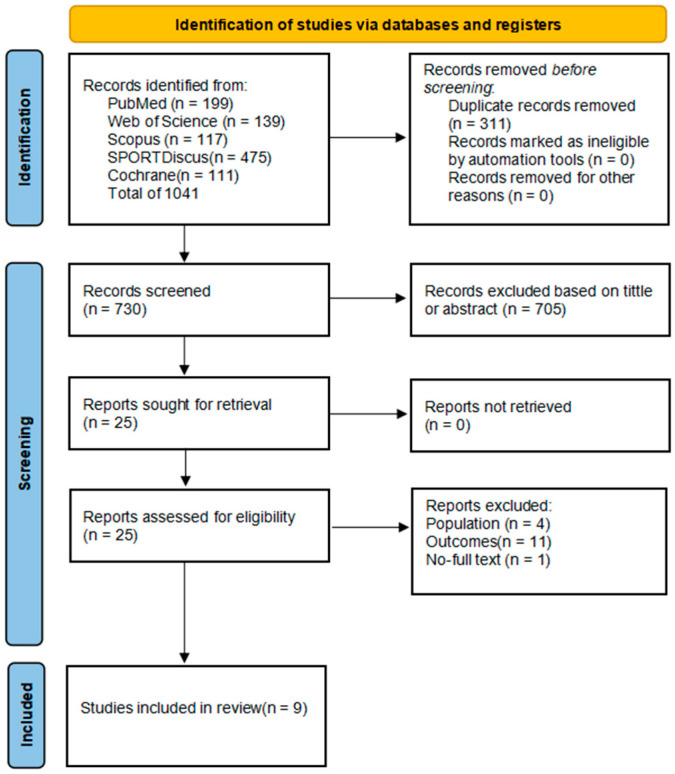
PRISMA Flow Diagram of Study Selection.

Subsequently, 25 full-text articles were retrieved for further assessment. After full-text evaluation, 16 studies were excluded—specifically, 4 due to inappropriate population characteristics, 11 for lacking relevant outcome data, and 1 due to the unavailability of full text (we attempted to contact the author without success, and the study did not appear to be peer reviewed). Ultimately, 9 randomized controlled trials (RCTs) met all inclusion criteria and were incorporated into the final analysis. The detailed study selection process is depicted in the PRISMA flow diagram (Figure 1).

### 3.2. Study Characteristics

#### 3.2.1. Participants

This systematic review included nine randomized controlled trials involving a total of 114 healthy female participants aged between 18 and 30 years (Table 1). The participants ranged from recreationally active women to trained athletes, including team-sport players, semi-professional rugby athletes, and elite performers in sports such as field hockey and water polo [49,52,67]. Two studies included both sexes but reported sex-specific outcomes, and was thus eligible [54,68]. All participants were free from chronic diseases and reported no medical contraindications to physical activity or supplementation.

#### 3.2.2. Intervention

All studies employed acute supplementation protocols using beetroot juice (BRJ) as the source of inorganic nitrate. The nitrate dose ranged from approximately 6.4 to 26 mmol, with supplementation typically consumed 2–2.5 h before testing. Placebo conditions were carefully matched in volume, taste, and appearance, using nitrate-depleted BRJ or alternative low-nitrate juices such as blackcurrant. The studies followed double-blind, crossover or parallel-group designs, and participants were instructed to maintain consistent dietary and training habits throughout the trials.

#### 3.2.3. Outcome Measures

The studies evaluated a wide range of sport performance outcomes, including muscle strength, power, and sprint capacity. Key indicators involved countermovement jump (CMJ) height and peak power, isometric maximal voluntary contraction (MVC), rate of torque development (RTD), and repeated sprint ability (RSA). Sprint tests ranged from 10 m to 30 m distances. Some studies also assessed muscular endurance (e.g., repetition to failure), recovery torque, and perceptual variables such as the rating of perceived exertion (RPE). Performance was typically measured using pre-post comparison designs with objective instruments such as force platforms, isokinetic dynamometers, and photoelectric timing systems.

### 3.3. Risk of Bias Assessment

Risk of bias was evaluated using the Cochrane RoB 2.0 tool across five domains. Six of the nine included studies were judged as having an overall low risk of bias. The remaining three studies [50,54,67] were rated as “some concerns,” mainly due to unclear randomization procedures or insufficient reporting of prespecified outcomes.

**Figure 2:**
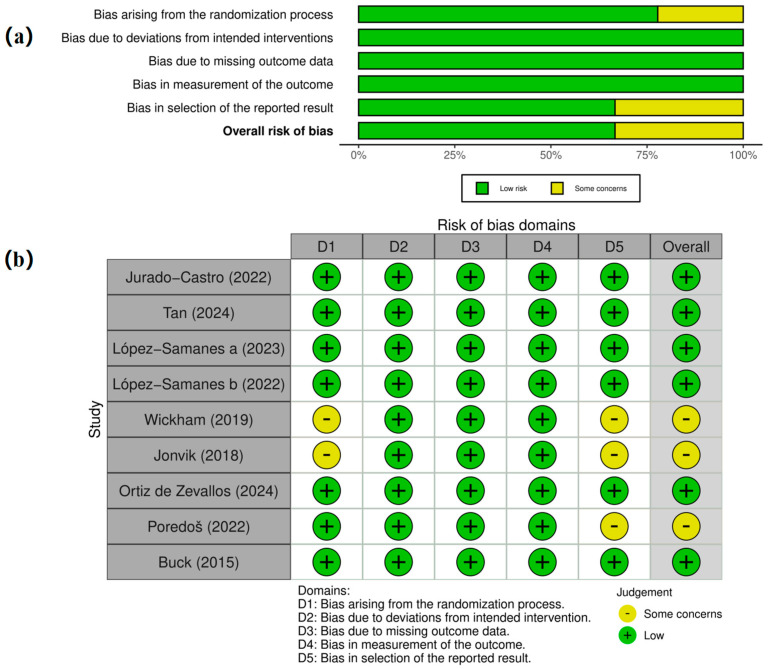
Risk bias assessment. (**a**) Summary plot of risk of bias across five domains for all included randomized controlled trials. (**b**) Risk of bias assessment for each individual study.

Domains related to intervention adherence, outcome measurement, and missing data were consistently rated as low risk across all studies. However, selective reporting (Domain 5) showed greater variability. Overall, the methodological quality of the included trials was acceptable and did not indicate high risk in any domain (Figure 2).

### 3.4. Effects on Performance Outcomes

All forest plots report standardized mean differences (SMD, Hedges’ g) with 95% confidence intervals (CI). Squares represent study-specific effect sizes, and the diamond represents the pooled effect size based on a random-effects model (Hartung–Knapp adjustment). Statistical significance was set at *p* < 0.05, and heterogeneity was assessed using the *I*^2^ statistic.

#### 3.4.1. Power

**Figure 3:**
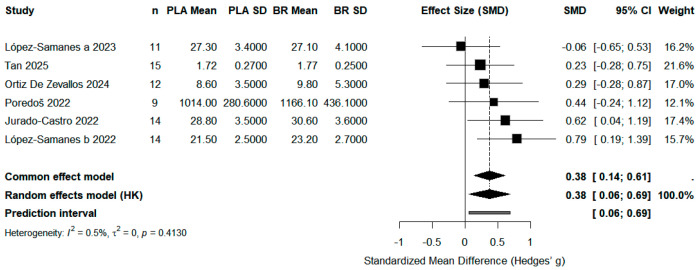
Effect of Beetroot Juice Supplementation on Power. Values are standardized mean differences (SMD) with 95% confidence intervals (CI). Positive values favor beetroot juice (BR) over placebo (PLA).

Six studies (*n* = 75) assessed power using countermovement jump height and peak lower-limb power. The pooled effect size favored beetroot supplementation with a moderate, statistically significant effect (SMD = 0.38, 95% CI: 0.06 to 0.69, *p* < 0.05), with very low heterogeneity (*I*^2^ = 0.5%). These findings indicate that acute beetroot juice supplementation can meaningfully enhance lower-body power performance in women (Figure 3).

#### 3.4.2. Muscle Strength

A total of 6 studies (*n* = 73) evaluated the effect of beetroot juice supplementation on muscle strength outcomes (e.g., isometric MVC, handgrip strength). The pooled result showed a small, non-significant effect in favor of nitrate supplementation (SMD = 0.10, 95% CI: −0.10 to 0.30, *p* > 0.05), with no heterogeneity detected among studies (*I*^2^ = 0%).

**Figure 4:**
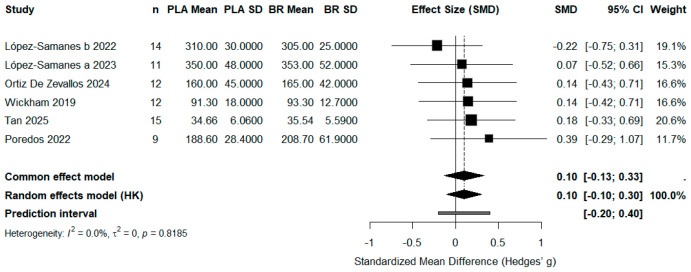
Effect of Beetroot Juice Supplementation on Muscle Strength. Values are standardized mean differences (SMD) with 95% confidence intervals (CI). The pooled effect was small and not statistically significant (*p* > 0.05).

These findings suggest that acute nitrate intake does not substantially improve muscle strength in female participants (Figure 4).

#### 3.4.3. Sprint Performance

Five studies (*n* = 67) examined the impact of nitrate supplementation on sprint performance (e.g., 10 m/20 m sprint time, intermittent sprint test). The overall pooled effect was small and not statistically significant (SMD = 0.14, 95% CI: −0.13 to 0.41, *p* > 0.05), with no heterogeneity (*I*^2^ = 0%).

**Figure 5:**
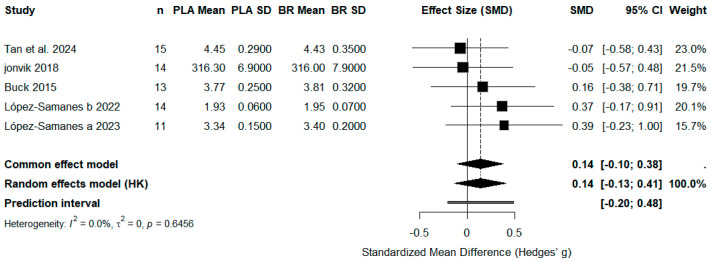
Effect of Beetroot Juice Supplementation on Sprint Performance. Values are standardized mean differences (SMD) with 95% confidence intervals (CI). The pooled effect was small and not statistically significant (*p* > 0.05).

These findings provide limited evidence for an ergogenic benefit of acute nitrate intake on sprint performance in female athletes (Figure 5).

### 3.5. Sensitivity Analysis

**Figure 6:**
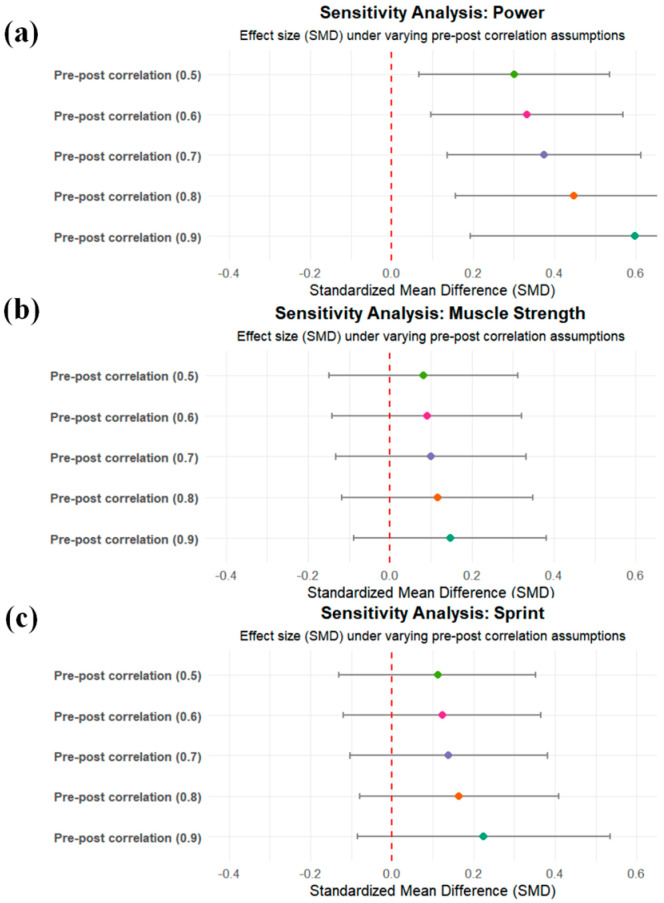
Sensitivity analyses under varying assumptions of pre–post correlation (r = 0.5 to 0.9) for: (**a**) muscle strength, (**b**) explosive power, and (**c**) sprint performance.

To examine the robustness of the pooled effect sizes to variations in the assumed pre–post correlation coefficient, sensitivity analyses were conducted across five r values (0.5 to 0.9) for each performance domain (Figure 6). Results demonstrated that the direction and magnitude of the standardized mean differences (SMDs) remained relatively stable across correlation assumptions, indicating that the main findings were not substantially influenced by the choice of *r*.

For muscle strength, SMD values remained relatively stable across different r assumptions (range: 0.05 to 0.14), indicating that the results were not sensitive to changes in r and can be considered robust.

For power, the primary analysis (*r* = 0.7) yielded an SMD of 0.38. When r increased from 0.5 to 0.9, the effect size increased progressively from 0.31 to 0.49, with all estimates remaining positive. This pattern suggests a consistent and robust ergogenic benefit of nitrate supplementation on power.

For sprint performance, the effect sizes showed minimal fluctuation across correlation scenarios (range: 0.10 to 0.21), with all estimates remaining in favor of nitrate supplementation. This consistency supports the reliability of the pooled estimates for sprint-related outcomes.

These findings collectively support the robustness of the meta-analytic results across plausible assumptions of within-subject correlations, reinforcing the methodological appropriateness of using SMCC with a default r = 0.7.

### 3.6. GRADE Summary

The certainty of the evidence was assessed using the GRADE framework (Table 2). For muscle strength, the overall certainty was rated as low, downgraded due to imprecision stemming from relatively small sample sizes and 95% confidence intervals that included the null effect. For power, the certainty was rated as moderate, with a minor downgrade due to some inconsistency across studies, although heterogeneity (*I*^2^ = 0.5%) remained low. For sprint performance, the evidence was also rated as low, primarily due to imprecision, as several effect sizes were small and confidence intervals crossed zero. These GRADE ratings suggest that while some benefits of beetroot juice supplementation are evident, particularly in power, caution is warranted when interpreting the strength and sprint-related findings.

## 4. Discussion

This study represents the first systematic review and meta-analysis to synthesize existing research on the effects of inorganic nitrate (NO_3_^−^) supplementation on exercise performance specifically in female populations, with a focus on three key physical performance indicators: muscle strength, power, and sprint performance. The main findings indicate that inorganic nitrate supplementation has limited efficacy in improving muscle strength and sprint performance in females, with small effect sizes and confidence intervals crossing zero. In contrast, a relatively consistent positive trend was observed for explosive power outcomes. These findings help address the existing gender gap in sports nutrition research and provide a scientific basis for developing individualized supplementation strategies for female athletes in the future.

### 4.1. Physiological Mechanisms Underlying the Improvements in Power

The meta-analysis revealed a positive trend in explosive power outcomes—particularly countermovement jump (CMJ) height and peak power (Pmax)—following inorganic nitrate supplementation in females. To date, only four studies have examined the effects of nitrate supplementation on CMJ performance in women [31,49,51,52]. CMJ execution is characterized by a rapid rate of force development (RFD) over a brief contraction period. Accordingly, CMJ height is widely regarded as a reliable indicator of lower-limb power strength. From a physiological standpoint, nitrate supplementation appears to be most effective during the initial phase of muscle contraction, where rapid force generation is critical [41,69]. Evidence suggests that NO_3_^−^ may enhance calcium ion kinetics and excitation-contraction coupling, thereby improving the contractile function and RFD of type II fast-twitch muscle fibers [70]. The relatively low oxygen tension surrounding type II fibers may further promote the reduction of nitrite to nitric oxide, leading to enhanced local perfusion, fatigue resistance, and muscle fiber contractility—all contributing to improved overall exercise performance [38].

In addition, nitric oxide may enhance energy utilization by promoting capillary recruitment and improving oxygen extraction efficiency in skeletal muscle, which is particularly relevant for high-frequency, short-duration explosive movements [27,71]. Given that women tend to exhibit higher capillary density in skeletal muscle, this may facilitate local blood flow and oxygen diffusion, thereby expanding the physiological window of response to beetroot juice supplementation and enhancing its ergogenic potential [72].

### 4.2. The Effects on Muscle Strength and Sprint Performance

In contrast to the findings on power, the effects of NO_3_^−^ supplementation on muscle strength and sprint performance appear limited, with statistically non-significant outcomes and confidence intervals generally crossing the line of no effect. This uncertainty may stem from two main factors:

First, the ergogenic effect of nitrate on power in females may be attributed to its role in optimizing short-duration energy systems, particularly the phosphocreatine (PCr) system [34]. NO_3_^−^ supplementation has been shown to delay PCr depletion during high-intensity, short-duration efforts, thereby enhancing the efficiency of initial energy output [39]. However, tests of muscle strength—such as maximal voluntary contraction (MVC) or isokinetic peak torque—are characterized by brief maximal efforts, where energy provision is dominated by PCr turnover and the outcomes are largely determined by neural drive and muscle cross-sectional area [73]. The benefits of NO_3_^−^ on the PCr system may not substantially translate to these maximal strength outputs, as nitrate does not directly influence neural efficiency or hypertrophic adaptations. This may account for the modest and inconsistent improvements in strength outcomes [40]. Consistently, a recent umbrella review also reported that nitrate supplementation had no significant ergogenic effect on muscle strength [74].

Second, sprint performance is influenced by a wide range of factors—such as training status, tendon stiffness, neural conduction velocity, and intermuscular coordination—making it difficult to isolate the effect of supplementation. These factors may obscure the observable benefits or lead to high inter-individual variability in response [75]. Even improvements in CMJ do not necessarily translate into faster 10 m or 30 m sprint times. In sports involving repeated sprint efforts, such as team-based activities [52,53], performance is determined by a multifaceted set of capacities—including acceleration-deceleration ability, joint stability, core control, and reactive strength—well beyond the scope of isolated power alone.

### 4.3. Sex-Specific Mechanisms of Response

This study exclusively included female participants, addressing the prevailing male-centric bias in sports nutrition research.

While previous meta-analyses have supported the ergogenic effects of NO_3_^−^ on endurance and strength performance in males, physiological differences between sexes may limit the generalizability of these findings to female populations [27,32,34]. Women differ significantly from men in terms of hormonal profiles, skeletal muscle cross-sectional area, and muscle fiber composition (i.e., type I vs. type II fiber distribution)—all of which may influence the synthesis and utilization of nitric oxide within skeletal muscle tissue [25].

On the other hand, women may have lower baseline dietary intake of NO_3_^−^, which could enhance the relative benefit of supplementation. However, they may also be more susceptible to saturation effects, leading to a non-linear dose–response relationship [76]. Therefore, future research should prioritize the optimization of dosing strategies and investigate response variability with a sex-specific lens.

In addition to efficacy, safety considerations are also important in female populations. A few studies explicitly stated that no adverse events occurred during nitrate supplementation, whereas the majority did not provide specific information on adverse events or safety outcomes. This highlights the need for future trials to systematically monitor and report potential adverse events in order to better establish the safety profile of nitrate supplementation in female athletes.

### 4.4. Methodological Variability and Its Impact on Study Outcomes

The primary studies included in this review exhibited methodological heterogeneity, which may partially account for the inconsistent findings observed. First, there was considerable variation in the nitrate supplementation doses across studies, ranging from 6.4 to 12.8 mmol, and no established sex-specific dosing guidelines currently exist for female participants. Second, performance assessment methods lacked standardization—some studies evaluated CMJ height, while others measured power output or rate of force development—necessitating variable harmonization during meta-analysis. In addition, intervention durations varied from acute (single-dose) supplementation to short-term protocols (up to 3 days [54]), which could result in differing underlying metabolic effects. These variations contribute to statistical uncertainty in the meta-analysis and underscore the need for greater consistency in study design and transparent reporting in future research.

### 4.5. Assessment of Analytical Robustness and Certainty of Evidence

Sensitivity analyses demonstrated that varying the assumed correlation coefficient (r = 0.5–0.9) did not substantially alter the direction or statistical significance of the effect on explosive power, suggesting the robustness of the primary findings. According to the GRADE assessment, all three outcomes were rated as having “moderate” quality of evidence, primarily due to small sample sizes, wide confidence intervals, and moderate heterogeneity across studies. Consequently, this review provides moderately credible preliminary evidence that NO_3_^−^ supplementation may enhance explosive power in females. However, current evidence for its effects on muscle strength and sprint performance remains insufficient and warrants further high-quality investigation.

## 5. Practical Implications and Study Limitations

Although this review did not yield strong evidence supporting the efficacy of NO_3_^−^ supplementation across all performance domains, the preliminary findings provide a reasonable basis for the short-term use of beetroot juice as a priming nutritional strategy in female athletes prior to competition. Coaches and sports nutritionists are advised to tailor supplementation protocols according to the demands of the specific sport task—particularly those requiring high power output—using a dosage of approximately 6–12 mmol NO_3_^−^ ingested 2–3 h before activity, with individualized adjustments based on subjective tolerance and response. It is also important to note that NO_3_^−^ should not be considered a substitute for long-term training. Instead, it should be integrated alongside resistance training or neuromuscular activation techniques to achieve sustained improvements.

Several limitations of this review should be acknowledged: (i) The total number of eligible studies was limited, and some pooled results were disproportionately influenced by individual studies; (ii) Although outcomes such as CMJ and Pmax can theoretically be categorized under power, aggregating these measures may obscure subtle differences in their specific effects; (iii) Most included trials did not report female-specific variables such as menstrual cycle phase or hormonal contraceptive use, which could influence nitrate metabolism. (iv) Only English-language publications were included, which may introduce language bias. (v) In studies using placebos other than nitrate-depleted beetroot juice, the phenomenon of “beeturea” (dark urine after beetroot intake) could have compromised blinding [77].

Future studies should employ larger sample sizes, longer intervention periods, and better control for menstrual and hormonal status to clarify the dose–response relationship, optimal timing of intake, and potential sex-specific mechanisms of action. In addition, future trials should evaluate the effectiveness of blinding, particularly in cases where placebos other than nitrate-depleted beetroot juice are used, to minimize the risk of performance or detection bias.

## 6. Conclusions

In summary, this review provides preliminary evidence that inorganic nitrate supplementation may enhance power in females, while its effects on muscle strength and sprint capacity remain inconclusive. Despite methodological heterogeneity, the findings support the potential use of nitrate as a short-term pre-competition strategy in female athletes. Further high-quality, sex-specific studies are needed to establish optimal dosing and clarify underlying mechanisms.

## Figures and Tables

**Table 1:** Characteristics of the Studies Included in the Systematic Review and Meta-Analysis of the Acute Effects of Beetroot Juice Supplementation in Women.

Study	Country	Population Description	BRJ Dose and Schedule	PL Dose and Schedule	Supplement Taking Time	Exercise Type	Training Details	Outcomes Measured Tool	Main Outcomes
Jurado-Castro (2022) [31]	Spain	14 Physically active women (Age: 25.36 ± 3.97 years)	70 mL Beetroot juice (400 mg nitrate)	70 mL blackcurrant juice (nitrate-depleted juice)	2 h pre-exercise	Resistance Training	3 sets × 3 exercises (75% 1RM), squat/leg press/leg ext. to failure	MyJump2, Speed4Lift, 75% 1RM test, RPE	CMJ, endurance reps, RPE
Tan (2024) [51]	USA	15 Trained team-sport athletes (Age: 20 ± 1 years)	Beetroot Juice (12.0 mmol NO_3_^−^)	Beetroot Juice (0.1 mmol NO_3_^−^)	2.5 h before test	Mixed: Sprint, Jump, Yo-Yo Test	NR	Timing gates, jump mat, handgrip dynamometer, Borg scale, Stroop test	Sprint (10 m, 20 m), CMJ, Handgrip, Ball throw, RPE, Cognitive flexibility, Oral microbiota
López-Samanes a (2023) [52]	Spain	11 Elite female hockey athletes (Age: 18.0 ± 1.0 years)	70 mL Beetroot Juice (6.4 mmol NO_3_^−^)	70 mL Beetroot Juice (0.04 mmol NO_3_^−^)	2.5 h before test	Field hockey	Match simulation: 2 × 12.5 min; 1-week washout between trials	Jump mat, handgrip dynamometer, sprint test, GPS tracker	CMJ, Handgrip, 20 m sprint, Repeated Sprint Ability, Match GPS data
López-Samanes b (2022) [49]	Spain	14 Semi-professional rugby women (Age: 25.0 ± 3.7 years)	Beetroot Juice (140 mL, 12.8 mmol NO_3_^−^)	Beetroot Juice (Placebo, 0.08 mmol)	2.5 h before testing	Rugby	Field test Within-subject, 2 visits	Jump mat, handgrip dynamometer, sprint test, GPS tracker	CMJ, isometric grip, 10/30 m sprint, agility test, Bronco test, RPE, side effects
Wickham (2019) [50]	USA	12 Recreationally active females using hormonal contraceptives (Age: 21.8 ± 3.4 years)	Beetroot juice (280 mL/day, ~26 mmol NO_3_^−^)	nitrate-free placebo	2.5 h before testing	cycling, plantar flexor torque	Cycling test	Plantar flexor torque	MVC, voluntary activation, low-frequency torque, RPE, HR
Jonvik (2018) [67]	Netherlands	14 Elite female athlete (Age: 22 ± 4 years)	4.8 g/day, divided into 3 doses	Beetroot juice (140 mL/day, ~800 mg nitrate)	Placebo (140 mL/day, 0 mg nitrate)	Water polo	Dynamic apnea test, intermittent swim sprint test	NA	Intermittent Sprint Test
Buck (2015) [53]	UK	13 Team-sport female amateur (Age: NR)	Beetroot juice (500 mL, ~8.4 mmol NO_3_^−^)	Placebo (NO_3_^−^-depleted)	2.5 h prior	Team sports	Simulated team-game circuit with repeated sprint sets; ~15 min × 4	Timing gates	Total sprint time, best sprint time
Ortiz de Zevallos (2024) [54]	USA	12 Recreationally active female(Age: 24 ± 4 years); 14 Recreationally active male(Age: 23 ± 4 years)	Beetroot juice (13 mmol NO_3_^−^)	NO_3_^−^-depleted beetroot juice, identical	2 h before	Isokinetic knee extension	30 repetitions at 180°/s; single bout sessions; Tested both sexes separately, matched for physical activity level	NA	Isokinetic peak power, MVC, recovery torque
Poredoš (2022) [68]	Slovenia	9 Female and 9 male Recreational athletes (Age: 29.1 ± 6.6 years);	Beetroot Juice (140 mL, ~12.8 mmol NO_3_^−^)	Placebo (140 mL, low-nitrate (<0.1 mmol NO_3_^−^))	2.5 h prior	Resistance Training	Seated knee extension test	Isometric dynamometer	MVC, RTD, isometric endurance at 50% MVC

Note: BRJ: Beetroot juice; PL: placebo; CMJ: countermovement jump; RPE: Rating of perceived exertion; MVC: Maximum voluntary strength; RTD: Rate of torque development; NR: Not report.

**Table 2:** GRADE level of evidence for this study’s findings.

Outcome	Total Participants (Studies)	Risk of Bias	Inconsistency	Indirectness	Imprecision	Other Considerations	Effect Estimate (Hedge’s g, 95% CI)	Certainty of Evidence (GRADE) *
Muscle Strength	73 (6 RCTs)	Not Serious	Not serious	Not Serious	Serious	Possible publication bias	0.10 [−0.10, 0.30]	⨁⨁◯◯ Low
Power	75 (6 RCTs)	Not Serious	Moderate	Not Serious	Not Serious	Possible publication bias	0.38 [0.06, 0.69]	⨁⨁⨁◯ Moderate
Sprint Performance	67 (5 RCTs)	Not Serious	Not serious	Not Serious	Serious	Possible publication bias	0.14 [−0.13, 0.41]	⨁⨁◯◯ Low

* GRADE interpretation of the certainty of evidence: ⨁⨁⨁◯ Moderate: We are moderately confident in the effect estimate; the true effect is likely to be close but may be substantially different. ⨁⨁◯◯ Low: Our confidence in the effect estimate is limited; the true effect may be substantially different.

## Data Availability

Data supporting the findings of this study can be requested from the corresponding author (J.L.) upon reasonable justification.

## Supplementary Materials

The following supporting information can be downloaded at: https://www.mdpi.com/article/10.3390/life15091425/s1, Supplementary Table S1: PRISMA Checklist; Supplementary Table S2: Search Strategy.
